# Family, Individual, and Other Risk Factors Contributing to Risk of Substance Abuse in Young Adults: A Narrative Review

**DOI:** 10.7759/cureus.32316

**Published:** 2022-12-08

**Authors:** Mustafa Alhammad, Rajeh Aljedani, Mohammed Alsaleh, Nawaf Atyia, Mohammed Alsmakh, Ali Alfaraj, Alya Alkhunaizi, Jalal Alwabari, Mohammed Alzaidi

**Affiliations:** 1 Internal Medicine, King Abdulaziz University Hospital, Jeddah, SAU; 2 Medicine, King Abdulaziz University Hospital, Jeddah, SAU

**Keywords:** drug addiction, personality traits, risk factors, substance abuse side effects, substance abuse

## Abstract

Substance use disorder and the availability of certain over-the-counter drugs are worldwide issues that affect many individuals, both mentally and physically. As a result, the frequent use of this substance can lead to substance abuse. This phenomenon is also becoming more prevalent with time, and it does not differentiate between genders, ages, races, or religions. This review aimed to provide an overview of studies related to substance abuse, the individuals who tend to abuse these substances, and their risk factors. We also aimed to discuss, identify, and analyze the factors that increase the risk of substance abuse among young adults. We performed a thorough search for related studies using PubMed to provide a comprehensive review of the risk factors and side effects experienced by young adults. The selected indexing terms included “substance abuse,”“risk factors,” and “personality traits,” among others. Information was gathered from relevant peer-reviewed publications, and thereafter refined, and summarized.

## Introduction and background

Substance use disorders refer to the recurrent use of alcohol and/or drugs causing significant clinical and functional impairment; these impairments can include health issues, disability, and failure to realize major responsibilities at work, school, or home. “According to the DSM-5, a diagnosis of substance use disorder is based on evidence of impaired control, social impairment, risky use, and pharmacological criteria” [1].

Substance abuse is a serious problem faced by diverse societies worldwide; this problem does not discriminate in terms of gender, age, race, or religious affiliation, which indicates that substance abuse is somehow associated with human nature in general [2]. “In 2016, the percentage of individuals between the ages of 15 and 64 years who abused substance at least once in their lifetime was approximately 5.6% globally.” During the last decade, the issue of substance abuse has multiplied among adolescents and young adults, especially among men aged 15-30 years. The Global Burden of Disease Study in 2013 showed that the maximum usage of these substances occurred among individuals aged between 18 and 25 years with the peak age of initiation falling between 16 and 18, and using these substances also causes 14% of health problems among young men [3,4].

Ethnicity-related disparities in drug usage, and its effects, have long been documented; however, the role of genetic and societal factors is still unclear. Even though racial healthcare inequalities are, in reality, the result of social and historical factors, they may be wrongly attributed to genetic variances. Drug use disorders are inherited illnesses affected by a complex interaction of genetic and environmental variables [5].

Existing research showed that annually, 11.8 million people die because of alcohol abuse. Cannabis is the most used illicit drug globally, with an estimate of more than 200 million abusers documented [6].

The use of prescription and over-the-counter (OTC) medications for non-medical purposes is a major area of concern. According to the Centers for Disease Control and Prevention, 20.2% of high school students misused prescription medicine in 2009. Drugs such as Vicodin, Oxycontin, Adderall, and Ritalin are among the prescription medications that are frequently abused. The latter two are typically used to treat attention deficit and hyperactivity disorder. Two more medicines that are frequently used for non-medical purposes are tranquilizers and cough medications. According to the National Institute on Drug Abuse, prescription and OTC medicines are the illicit substances that twelfth-grade students abuse most frequently [7].

An individual who abuses substances is susceptible to a lower quality of life than a healthy non-abuser in multiple aspects and could experience psychological, physical, social, educational, and functional impairments. Research studies show that in the United Kingdom, problematic Internet use is associated with a wide range of negative social and psychological effects, including depression, bullying, alcohol and drug abuse, and depression [8,9]. Moreover, any health issue could double in severity when abusers use two or more of psychoactive substances, compared with abusers who use a single drug. These individuals will also be exposed to a much higher degree of possible injury, poor educational level, violence, depressive symptoms, dangerous driving, or suicidal thoughts and attempts. In certain age groups, such as adolescence and young adulthood, polysubstance abuse can have multiple serious consequences that could affect their future, especially because this age is considered to be a transition period from adolescence to adulthood. Drug abuse can affect the future of their social relationships, identity development, and educational advancement [9]. Furthermore, due to possible risky conduct, such as sharing needles, substance use increases the risk of HIV infection, while actual drug use could worsen actual HIV/AIDS-related health problems [7]. Considering the above, this review aims to identify, discuss, and analyze the risk factors that can result in young adults’ substance abuse.

## Review

Review-risk factors

History of Abuse in Childhood

A study conducted at a clinic treating patients with substance abuse evaluated 105 patients with significant histories of domestic violence; of these patients, 14% reported being a victim of childhood abuse [10]. Similarly, a cross-sectional study surveyed mothers with children who were six years old and younger. Of the 733 respondents, 36% reported a history of substance abuse; this positive response was more common among those who had a history of childhood abuse [11]. Furthermore, children with a history of abuse have more serious substance abuse issues later in their adulthood; this conclusion was extracted from a retrospective study involving 643 patients [12].

Family History of Substance Abuse

Regarding the impact of family history on an individual’s future risk of devolving into substance abuse, one study discussed substance abuse among medical students and doctors. The results showed that a family history of substance abuse was among the risk factors that made individuals prone to misuse any subtype of substance [13]. Moreover, a study on methadone maintenance therapy and positive parental history concluded that patients with a positive family history had more opioid dependence symptoms that are so severe that they can be classified as severely dependent [14].

Individual Risk Factors

There are multiple individual risk factors for substance abuse that can be categorized by age group. For example, children and adolescents younger than 18 years old try substances for several reasons; to have a new experience or for adventure, interpersonal trauma, ethnicity, gender, and socioeconomic status. Among young adults between 18 and 25 years of age academic stress plays a major role in substance abuse, as well as the long-term use of prescription medications after minor surgery, or a poor relationship with parents. Adults aged 26 to 64 years often face major life challenges and must maintain a balance between job and family life, which tends to increase the risk of substance abuse, especially if they are in a high-stress profession, such as lawyers, healthcare workers, or military personnel. Experiencing grief, or the loss of a close member of a family or friend can lead to substance abuse. Lastly, adults who are older than 65 years face several issues, such as grief, chronic diseases, lack of care, and social isolation, that could lead them to use drugs in inappropriate doses [15].

Peer Risk Factors

Many factors affect substance abuse, such as loss of family support, friends' influence, and other individualized factors. Peer factors are some of the strongest factors, that influence individuals to start abusing substances. An Iranian study published in 2014 interviewed people with drug addiction and found that peers have a great influence on drug use. However, individuals who join their peers in using illegal substances do not do so by accident; many factors draw these individuals toward friends or peers, who abuse substances, such as family issues, or behaviors and attitudes that they have in common with these groups. Often, people seek out the safety that these groups or gangs provide. However, peer factors are not just about influencing others to use illicit substances; they could also influence a person to start smoking and drinking alcohol when socializing, which could, in turn, lead to the use of illegal drugs [16].

Environmental Risk Factors in Childhood and Adolescence

According to a study conducted in 2015, a person’s environment is one of the factors that significantly influences their risk of addiction [17]. A total of 600 respondents categorized as urban and rural were interviewed. It was found that several risk factors, including being male, having friends who are a “bad influence,” the parental norm, fathers’ and mothers’ educational level, and parental connectedness, were associated with poor outcomes in adults’ lives [18].

Co-occurring Disorders

A dual diagnosis is defined as a combination of disorders such as severe mental illness and substance abuse disorders [19]. There are many mental health comorbidities that can affect substance abuse; for example, anxiety disorders, depression, bipolar disorder, attention deficit hyperactive disorders, psychotic illnesses, borderline personality disorders, schizophrenia, antisocial personality disorder, emotional disorders, and other psychotic mental illnesses, that could lead to serious mental illness, and eventually cause substance abuse disorders [19]. In the United States, 7-10 million people have dual diagnoses of severe mental illness and substance use disorder. Moreover, most of these people were diagnosed with a mental illness in an early phase of their life, with substance use disorder following later [20]. A Spanish study conducted at a private hospital for addiction treatment showed that 798 (33.8%) out of 2,361 participants had mental illnesses, with depression being the most common disease combined with substance abuse, followed by anxiety disorders. These conditions had many risk factors including divorce, widowhood, high educational level, and being female. In terms of substances, alcohol has the highest rate of substance abuse (48.5%) in a dual diagnostic setting, with opiates being at the lowest end of the spectrum (27.4%) [21].

Genetic Polymorphism

Environmental and genetic variables have both been associated with addiction. Determining how genetic variables contribute to the etiology of addiction may enhance patient responsiveness to care and aid in disease prevention. By identifying the genes that are essential for neuroadaptation, both genome-wide approaches and candidate gene studies have been used to investigate the fundamental significance of the genetic component of drug addiction [22]. There are multiple studies that support the link between genetic polymorphism and substance abuse. One such study involved male participants from Jordan of Arab heritage. Nearly 498 individuals were determined to be addicted to one or more of various substances, including synthetic cannabinoids (47.5%), cannabinoids (19.6%), amphetamine (5.7%), alcohol (5.5%), benzodiazepines (4.6%), opiates (4.4%), cocaine (1.1%), and cannabis (0.4%). In 89% of the cases, only one drug was used. However, 11% of the participants with addiction in this study used multiple drugs [13]. The study found that the OPRM1 gene’s rs1799971 was associated with drug addiction, for both alleles and genotypes. Further, the age of addicted individuals, their smoking, and their marital status may be associated with the risk of developing a drug addiction, along with genetic variants in the OPRM1, OPRD1, and OPRK1 genes [22]. Another study on the brain-derived neurotrophic factor (BDNF), a member of the neurotrophic factor family, shows that it may be involved in the mechanisms behind substance misuse, according to data from animal research and genetic tests on humans. The BDNF-gene Val66Met polymorphism’s potential link to substance misuse was investigated in a polymorphism study involving 122 healthy individuals, 200 heroin users, and 103 methamphetamine users. It was thought that there might be a link between this polymorphism and the age of onset of drug addiction, in cases of heroin dependence. A decrease in the 66Met carrier frequency was linked to substance misuse, as can be seen by the significant variations in BDNF Val66Met genotype distribution between persons dependent on methamphetamine (p= 0.046) or heroin (p= 0.045) and healthy controls. In addition, compared to Meet allele carriers, the heroin-dependent group’s Val/Val homozygotes experienced a slower start of substance misuse. These findings imply that the pathophysiology of substance misuse may include the BDNF Val66Met polymorphism or a neighboring location. The results confirm earlier genetic scan findings suggesting that BDNF may increase susceptibility to substance misuse [23]. Major and minor allele frequency data are available in PubMed; however, there are no data available on population prevalence. In the following populations, SNPedia provides population diversity percentages for homozygous SNP, homozygous normal, and heterozygous, for all but one of our SNPs (rs4532; rs1800497; rs6280; rs1800955; rs4680 and rs1799971). There are unfortunately no statistics on the population prevalence of dinucleotide or a variable number of tandem repeats [24]. Almost all previous studies suggest a significant link between genetic polymorphism and drug abuse, with a wide variety of gene involvements [22-24].

Misuse of Prescription or OTC Medicine

Recently, there have been increasing reports of the misuse of prescription and OTC drugs. OTC drugs are medications available without supervision, meaning that people can easily procure them. They are typically safe if used in recommended and prescribed doses. However, these drugs can also be harmful, as overdose can lead to addiction and dependency [25]. Moreover, they also pose a significant risk of organ damage [25,26]. A cross-sectional descriptive observational study targeting the entire Spanish population was conducted using an online questionnaire. The results showed that 78.9% of the participants had previously taken or were currently taking OTC drugs. This consumption decreased as the age of the participants increased, with only approximately 36.4% of them aged ≥ 71 taking OTC drugs [27].

History of Substance Use

Prior history of substance abuse plays a major role in developing addiction in the future. This is evidenced by several studies, stating that alcohol and drug use prior to adolescence is a risk factor for developing substance abuse disorder later in life. Moreover, alcohol use at various initiating ages has been found to be associated with the risk of addiction and abuse, and one study concluded that an initiation age of drinking alcohol younger than 11 years old tends to lead to adult alcohol abuse [28]. Furthermore, the use of tobacco early in life and continuing to use tobacco during adolescence has been linked to a high risk of future cannabis and alcohol abuse [29,30].

## Conclusions

The likelihood of acquiring substance misuse later in life is significantly impacted by a history of substance abuse, this was evident in several research studies. According to a study, drinking alcohol before the age of 11 has the propensity to cause chronic adult alcoholism. Early cigarette smoking has also been linked to a significantly increased risk of later developing drug and alcohol abuse.

Various risk factors have not only been associated with but have also negatively influenced rehabilitation measures. This review found a correlation between the history of childhood abuse and individuals’ substance abuse; moreover, these individuals often experience serious substance abuse issues later in adulthood.

During our review of the risk factors, we also found that a family history of substance abuse is one of the risk factors evidenced by a study involving medical students and doctors suffering from substance abuse and another study on methadone maintenance therapy and a family history of substance abuse.

Substance abuse is affected not only by individuals’ environments or backgrounds but also by other factors such as age, interpersonal trauma, ethnicity, gender, academic stress for those aged 18-25 years, long-term use of prescribed medication, and socioeconomic status. Further, being male and having friends or peers who are a “bad influence” and even the educational level of both parents have an impact on an individual's substance abuse, particularly their behavior around their child. Also, substance abuse can result from grieving the loss of a close family member or friend.

The coexistence of a mental disorder may also lead to the misuse of substances, considering the vulnerability of these individuals’ mental state; this conclusion is evidenced by a study from the United States, which showed that 7 to 10 million individuals with substance abuse disorders have coexisting mental health disorders. Another study conducted in Spain found that the most common comorbid mental health disorder was depression, followed by an anxiety disorder.

Genetics has also been shown to play a role in addiction; this is evidenced by a study conducted in Jordan in which they discovered that the OPRM1 gene is implicated in drug addiction. Additionally, another study investigating BDNF showed that it may be involved in the mechanism of addiction or substance misuse. Prescription or OTC medications, which are the easiest and most accessible drugs to individuals, are also often abused. Although these drugs are safe, they are dangerous if the recommended doses are exceeded, which may lead to dependency and addiction.

In conclusion, this narrative review study has revealed that substance abuse drastically affects an individual, both clinically and functionally, often causing significant impairment. Encouraging the next young generations to control their bad habits may benefit in preventing further self-harm. Further large-scale and well-designed randomized controlled clinical studies that explore substance abuse effects are necessary.
